# Are Transperineal Ultrasound Parameters Useful to Predict Incontinence in Patients with Single-Incision Mini-Slings?

**DOI:** 10.3390/tomography8050213

**Published:** 2022-10-12

**Authors:** José Antonio García-Mejido, Pedro Blasco-Hernandez, Cristina Fernandez-Conde, Sara García-Pombo, Ana Fernández-Palacín, Carlota Borrero, José Antonio Sainz-Bueno

**Affiliations:** 1 Department of Obstetrics and Gynecology, Faculty of Medicine, University of Seville, CP 41004 Seville, Spain; 2 Department of Obstetrics and Gynecology, Valme University Hospital, CP 41014 Seville, Spain; 3 Department of Urology, Valme University Hospital, CP 41014 Seville, Spain; 4 Biostatistics Unit, Department of Preventive Medicine and Public Health, University of Seville, CP 41004 Seville, Spain

**Keywords:** 3D transperineal ultrasound mid-urethral sling, ultrasound, mesh complications

## Abstract

It would be logical to think that single-incision mini-slings (SIMS) should behave like the rest of the tension-free vaginal tape and, therefore, to believe that they present a similar ultrasound appearance, but there are no studies on this matter. Therefore, the main aim of our research is to determine which ultrasound parameters are associated with stress urinary incontinence (SUI) in patients carrying SIMS. A prospective observational study was carried out including 94 patients who were candidates for SUI corrective surgery with SIMS between 1 January 2021 to 31 December 2021 at the Universitary Hospital of Valme (Seville, Spain). A transperineal ultrasound evaluation was performed (six months after surgery) in order to study: the bladder neck–symphyseal distance, the posterior urethro–vesical angle, the pubic symphysis–tape gap, the tape–urethral lumen distance, the sagittal tape angle, the tape position, the concordance of movement between the tape and the urethra, and the axial tape angle. A total of 92 patients completed the study (63 asymptomatic and 29 symptomatic). Statistical differences were observed in the concordance of movement between the tape and the urethra (84.1% vs. 25.0%; *p*: 0.001) and in the axial tape angle at rest (139.3 ± 19.0 vs. 118.3 ± 15.4; *p*: 0.003) and at Valsalva (145.1 ± 20.2 vs. 159.1 ± 9.0; *p*: 0.034). Sagittal tape angle at rest was higher in urge urinary incontinence (UUI) patients (132.5 ± 35.7 vs. 143.3 ± 29.8; *p*: 0.001) and mixed urinary incontinence (MUI) patients (132.5 ± 35.7 vs. 157.8 ± 23.6; *p*: 0.025) compared to asymptomatic patients. In conclusion, the concordance between the movement of the tape and the urethra is the most useful ultrasound parameter to define continence in patients with SIMS.

## 1. Introduction

Stress urinary incontinence (SUI) affects a large number of patients, causing a severe impact on their quality of life. This has led to an increase, over the past decades, in the appearance of surgical procedures for its correction. The main surgical technique established for the treatment of SUI is the placement of a mid-urethral sling (MUS) in a retropubic or a transobturator position. Notwithstanding the previous, a new type of device called single-incision mini-slings (SIMS) has recently emerged, and the technique consists in anchoring it to the obturator musculature without passing through it [1].

At the same time, ultrasound has been integrated in the post-surgical analysis of patients operated with MUS, showing its anatomical location both statically and dynamically [2]. The importance of ultrasound in the evaluation of MUS relies on defining its position and its behavior during the Valsalva maneuver, and the correlation with symptoms and post-surgical complications [3,4,5]. Much research has been published, aiming to elucidate the mechanism of action of tapes; most of this research was performed in postoperative patients with tension-free vaginal tape (TVT)**,** trans-obturator tape (TOT), and transobturator tension-free vaginal tape (TVT-O) [6,7,8,9,10,11,12]. In these studies, different criteria has been evaluated: the compression exerted by the TOT on the urethra [6], the tape migration rate [7], the angulation between the two arms of the mesh [7], the position of the tape regarding the pubis [8,10] or its location with respect to the urethra [12], and the mobility and funneling of the bladder neck [11]. There is very little published on the ultrasound assessment of SIMS: on the one hand, it would be logical to think that SIMS should behave like the rest of the tapes (TOT, TVT and TVT-O) and, therefore, to believe that they present a similar ultrasound appearance but, on the other hand, there are no studies on this matter. Therefore, the aim of our work is to determine which ultrasound parameters are associated with SUI in patients operated with SIMS.

## 2. Materials and Methods

### 2.1. Subjects

A prospective observational study was performed including 94 patients consecutively recruited from 1 January 2021 to 31 December 2021 at the Universitary Hospital of Valme (Seville, Spain). The study was approved by the Biomedical Ethics Committee of the Junta of Andalusia (0523-N-21).

### 2.2. Data Collection

Patients included in the study had to be candidates for SUI corrective surgery with SIMS and had to sign a written informed consent. Patients with corrective pelvic floor surgery were excluded. All patients preoperatively underwent a standardized interview, which included questions about stress urinary incontinence (SUI), urge urinary incontinence (UUI) or mixed urinary incontinence (MUI).

The clinical evaluation was based on an urogynecological examination, using the International Continence Society Pelvic Organ Prolapse Quantification (ICS POP-Q) system to assess pelvic organ prolapse [13] and SUI status (simple stress test). In those cases where the type of urinary incontinence was unclear, a urodynamic test was performed to reach a diagnosis.

### 2.3. Ultrasound Assessment

**Figure 1:**
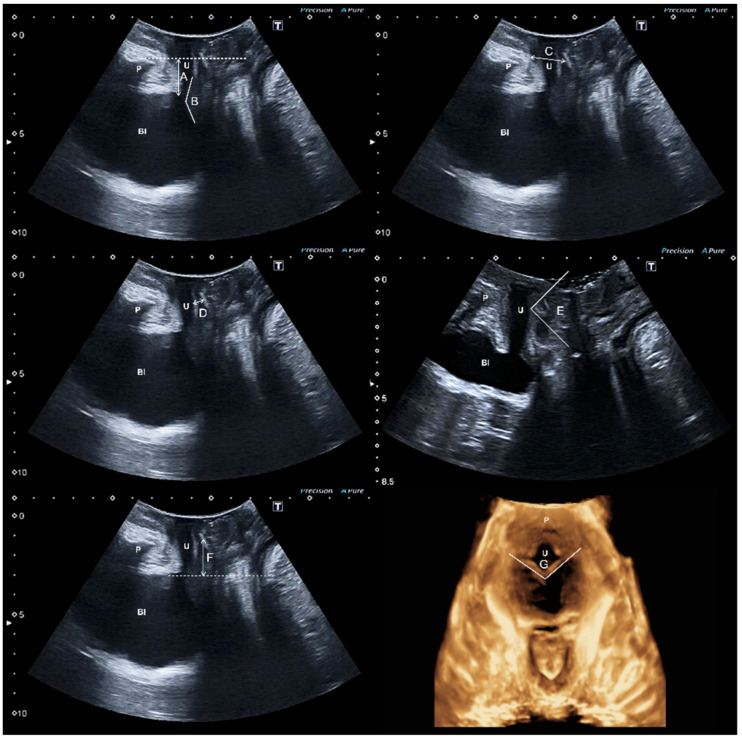
The ultrasound parameters included were the following: bladder neck–symphysis distance (A), posterior urethro–vesical angle (B), the pubic symphysis–tape gap (C), tape–urethral lumen distance (D), sagital tape angle (E), tape position (F), axial tape angle (G)m pubis (P), bladder (Bl), and urethra (U).

Post-surgical clinical analysis was performed six months after surgery and was similar to the one performed pre-surgically. The transperineal ultrasound evaluation was carried out by a single examiner (JAGM) with specific training in pelvic floor ultrasound. The examiner was unaware of the patients’ clinical data and used a 500^®^ Toshiba Aplio ultrasound machine (Toshiba Medical Systems, Tokyo, Japan) with a PVT-675MV 3D abdominal probe. The methodology followed during the transperineal pelvic floor ultrasound [14] consisted of capturing three volume measurements for each patient: at rest, during the Valsalva maneuver (minimum of 6 s [15]) and at maximum contraction, with a bladder filling volume of 200–300 mL estimated by transperineal ultrasound [4]. The ultrasound parameters included were the following (Figure 1):

Bladder neck–symphysis distance [16]: vertical distance from the bladder neck to the horizontal line passing through the posteroinferior margin of the pubic symphysis.

Posterior urethro–vesical angle [17]: angle between the urethral axis and the bladder floor wall next to the proximal third of the urethra.

The pubic symphysis–tape gap [6,18,19,20,21]: distance between the center of the sling and the lower edge of the pubic symphysis.

Tape–urethral lumen distance [4]: shortest distance between the tape and the urethral lumen.

Sagital tape angle [6]: Angle showing the tape in the midsagittal image.

Tape position [4,5,6,16,22,23,24,25,26,27]: positioning percentile of the midpoint of the tape regarding the total urethral length.

The concordance of movement between the tape and the urethra [5]: on the one hand, a concordant movement exists whenever the location of the tape at rest is identical to that during Valsalva; on the other hand, a discordant movement appears whenever there is a difference in the sling location between rest and Valsalva or if there is no contact between the tape and the urethra.

Axial tape angle [28]: Angle showing the tape in the axial image.

Levator hiatus area was studied in the plane of minimal hiatal dimensions [14] at rest and during the Valsalva maneuver. The integrity of the levator ani muscle (LAM) was assessed at maximum contraction using tomographic ultrasound imaging, as it has been previously described in literature [29,30]. Complete avulsion was diagnosed when an abnormal LAM insertion was observed in the three central sections. In unclear cases, a levator–urethra gap ≥ 2.5 cm was used to define an abnormal insertion [31].

### 2.4. Statistical Analysis

Quantitative variables were summarized using means and standard deviations (±SD) or, in the case of remarkably asymmetric distributions, with medians and 25th and 75th percentiles, and qualitative variables with percentages. Comparison of the numeric variables between the groups defined by the presence of incontinence (asymptomatic/symptomatic) was performed using Student’s *t*-test for independent samples or the nonparametric Mann–Whitney U-test in the case of abnormal distributions (studied using the Shapiro–Wilk test).

When the difference was significant, it was quantified using 95% confidence intervals. The statistical significance level was previously set in 95% (*p* < 0.005). Data analysis was performed with the statistical package IBM Corp. Released 2013. IBM SPSS Statistics for Windows, Version 22.0. Armonk, NY, USA: IBM Corp.

For the estimation of the statistical power, we relied on the study of Chantarson et al. [6], using the parameter pubic symphysis–tape gap, since it is one of the most studied parameters in urethral compression in patients wearing MUS [6,18,19,20,21]. To identify a difference in the gap between the pubic symphysis–tape of 1.5 mm between asymptomatic patients (distance 10.81 ± 1.05 mm to pubis [6]) and symptomatic patients (mean distance 12.29 ± 1.25 mm [6]), with an alpha error of 5% and a power of 80%, we had to recruit 12 patients for each group. The nQuery Advisor Release 7.0 program was used to calculate the sample size.

## 3. Results

A total of 94 patients were included: 2 patients did not attend the ultrasound assessment 6 months after surgery, so 92 completed the study (63 asymptomatic and 29 symptomatic).

The general characteristics of the study population, differentiating whether they presented SUI (symptomatic) or not (asymptomatic), after SIMS placement are shown in Table 1.

The body mass index (BMI) was higher in symptomatic patients with post-surgery urinary incontinence (28.4 ± 4.4 vs 30.7 ± 3.5; *p*: 0.015). The patients who remained asymptomatic after SIMS placement, pre-surgically presented SUI in 87.3% of the occasions and MUI in 12.7% of the cases, in contrast with the symptomatic patients, who pre-surgically presented SUI in 69.0% of the occasions and MUI in 31.0% of the cases (*p*: 0.046).

The parameters of the ultrasound evaluation performed 6 months after SIMS placement are shown in Table 2 and classified in two groups, according to the presence or absence of urinary incontinence (symptomatic or asymptomatic). No statistically significant differences were found in the ultrasound parameters studied between asymptomatic and symptomatic patients, except in the axial tape angle at rest (139.3 ± 19.0 vs. 129.8 ± 18.9: *p*: 0.029).

The variables of the ultrasound evaluations performed 6 months after SIMS placement, differentiating asymptomatic patients from patients with SUI, UUI and MUI are in Table 3. Statistical differences were observed in the concordance of movement between the tape and the urethra (84.1% vs. 25.0%; *p*: 0.001) and in the axial tape angle at rest (139.3 ± 19.0 vs. 118.3 ± 15.4; *p*: 0.003) and at Valsalva (145.1 ± 20.2 vs. 159.1 ± 9.0; *p*: 0.034). The sagittal tape angle at rest was higher in UUI patients (132.5 ± 35.7 vs. 143.3 ± 29.8; *p*: 0.001) and MUI patients (132.5 ± 35.7 vs. 157.8 ± 23.6; *p*: 0.025), compared to asymptomatic patients.

## 4. Discussion

Out of all the ultrasound parameters studied after surgery with SIMS, only the following presented statistical differences between asymptomatic patients and patients with SUI: the concordance of movement between the tape and the urethra (84.1% vs. 25.0%; *p*: 0.001), the axial tape angle at rest (139.3 ± 19.0 vs. 118.3 ± 15.4; *p*: 0.003) and at Valsalva (145.1 ± 20.2 vs. 159.1 ± 9.0; *p*: 0.034).

Tunitsky-Biton et al. [28] observed different measurements for axial tape angle among the various types of MUS studied. This suggests that this angle is specific of the type of MUS used, since it depends on the type of surgical technique performed [28]. What Tunitsky-Biton et al. [28] did not describe was the possible relationship that this axial tape angle could have with the presence or absence of post-surgical SUI.

We have observed that the axial tape angle is smaller in patients with SUI at rest. However, during Valsalva the opposite occurs, as SUI patients present a larger axial tape angle than asymptomatic patients. These findings suggest that the axial tape angle remains more stable between rest and Valsalva in asymptomatic patients than in patients with SUI.

Furthermore, in SIMS we were able to see that the concordance of movement between the tape and the urethra is an ultrasound parameter that distinguishes asymptomatic patients from patients with SUI. Previously, the concordance of movement between the tape and the urethra was described in all patients with optimal results and in 34% of patients with suboptimal results after TOT placement [6]. Nonetheless, we have observed that this concordance is present, but in a greater percentage of patients, possibly due to the anatomical variations of the different insertion points of the diverse tapes.

One of the most commonly used ultrasound parameters for the assessment of MUS is tape position. In fact, it has been reported that in incontinent patients after MUS placement the tape was located in the proximal third of the urethra [18,23,24,25], and that a successful surgical outcome was associated with a tape situated in the mid-urethra [5,22]. In contrast, Hueih Ling Ong et al. observed that asymptomatic patients could also have the tape placed in the proximal urethra [27]. It appears that localized tape position between 50–80% demonstrates a 91% success rate in patients with TVT [4]. However, this relationship between tape position and SUI has not been objectified in other studies [6,26], as occurs in our work with SIMS.

Two of the most commonly used measures of MUS compression in the literature are: the pubic symphysis–tape gap [6,18,19,20,21] and the tape–urethral lumen distance [4]. Although we do not have a cut-off point to define SUI in the gap between the pubic symphysis–tape, we should mention that patients with SUI present a larger pubic symphysis–tape gap than continent patients [6,18,19,20,21]. Measurements of this gap for continent patients range from 10.9 mm to 20.3 mm, and for SUI patients from 13.3 mm to 26.1 mm [6,18,19,20,21].

However, cut-off points have been established for the tape-urethral–lumen distance [4], determining measurements of 3–5 mm for continent patients, greater than 5 mm for patients with SUI and less than 3 mm for patients with obstructive symptoms [4]. In our study, we have not found differences in the pubic symphysis–tape gap and the tape–urethral lumen distance between asymptomatic and symptomatic patients.

Previous studies with other types of MUS define different measurements to determine SUI [4,6,16,18,19,20,21,22,23,24,25,26,27,28]. However, we have described that the concordance between the movement of the tape and the urethra is the most useful ultrasound parameter to define continence in patients with SIMS.

Our main strength lies in the fact that this is the first study focused on the post-surgical assessment of SIMS by ultrasound. In addition, we initially thought that SIMS would behave like any other MUS and we initially made a sample calculation based on what was previously established in the literature [6]. In spite of this, we have observed that the SIMS behavior differs from that of the rest of the MUS, which is why our data varies from what was initially expected. The classic ultrasound measurements used in previous publications about MUS are not useful for SIMS, being the concordance of movement between the tape and the urethra the most related variable to SUI in these patients.

Our main weakness lies in the fact that we have not clinically assessed the severity of SUI, not being able to relate the severity of urinary incontinence to ultrasound findings. Nor can we prove the influence of associated pathology, such as pelvic organ prolapse (POP), to the ultrasound findings of SIMS as we only count with a small number of patients with POP.

## 5. Conclusions

A concordant movement between the tape and the urethra is the most useful ultrasound parameter to define urinary continence in patients with SIMS. However, a discordant movement between the tape and the urethra is associated with SUI in patients with SIMS.

## Figures and Tables

**Table 1:** General characteristics of the population studied, differentiating asymptomatic from symptomatic patients after placement of the SIMS.

	Post-Surgical Clinical Diagnosis		*p*	IC 95%
	Asymptomatic (n: 63)	Symptomatic (n: 29)		
Age (years)	57.4 ± 8.8	58.2 ± 11.4	0.950	−5.0; 5.0
BMI	28.4 ± 4.4	30.7 ± 3.5	0.015	−4.1; −0.5
Deliveries	2.2 ± 0.9	2.3 ± 1.1	0.291	−1.0; 0
Menopause	38/59 (64.4%)	19/29 (65.5%)	1	−22.9%; 20.7%
Age at menopause (years)	49.5 ± 4.4	49.1 ± 3.8	0.919	−3.0; 3.0
Histerectomizada	3/63 (4.8%)	2/29 (6.9%)	0.649	−12.3%; 8.1%
Cystocele	4/63 (6.3%)	4/29 (13.8%)	0.436	−22.0%; 7.1%
Hysterocele	0/63 (0%)	1/29 (3.4%)	0.315	−10.5%; 3.6%
Rectocele	0/63 (0%)	1/29 (3.4%)	0.315	−10.5%; 3.6%
Enterocele	0/63 (0%)	0/29 (0%)	----	----
Elongation cervical	0/63 (0%)	0/29 (0%)	----	----
Pre-surgical clinical diagnosis				
SUI	55/63 (87.3%)	20/29 (69.0%)	0.046	0.01%; 37.0%
UUI	0/63 (0%)	0/29 (0%)		----
MUI	8/63 (12.7%)	9/29 (31.0%)		−37.0%; −0.01%
Presurgical Urodynamics				
Asymptomatic	4/52 (7.7%)	2/29 (6.9%)	0.543	−10.9%; 12.5%
SUI	44/52 (84.6%)	22/29 (75.9%)		−9.7%; 27.1%
UUI	1/52 (1.9%)	1/29 (3.4%)		−9.1%; 6.1%
MUI	3/52 (5.8%)	4/29 (13.8%)		−7.4%; 11.4%
Unrealized	11/63 (17.5%)	0/29 (0%)		8.1%; 26.9%

Body mass index (BMI); stress urinary incontinence (SUI); Urge urinary incontinence (UUI); Mixed urinary incontinence (MUI).

**Table 2:** Ultrasound evaluation of the study population differentiating asymptomatic from symptomatic patients after SIMS placement.

	Post-Surgical Clinical Diagnosis		*p*	IC 95%
	Asymptomatic (n: 63)	Symptomatic (n: 29)		
Bladder neck–symphyseal distance (rest)	23.8 ± 4.9	23.5 ± 6.9	0.846	−2.6; 3.2
Bladder neck–symphyseal distance (Valsalva)	15.8 ± 7.4	14.5 ± 7.9	0.444	−2.1; 4.7
Posterior urethro–vesical angle (rest)	118.5 ± 17.7	114.3 ± 13.9	0.509	−4.0; 9.5
Posterior urethro–vesical angle (Valsalva)	132.2 ± 25.0	130.5 ± 19.9	0.940	−12.0; 10.5
Gap between the symphysis pubis–tape (rest)	14.1 ± 4.3	14.2 ± 3.0	0.635	−1.8; 1.2
Gap between the symphysis pubis–tape (Valsalva)	12.8 ± 3.2	12.8 ± 3.4	0.656	−1.5; 1.0
Tape–urethral–lumen distance (rest)	3.1 ± 0.8	3.2 ± 1.1	0.778	−0.5; 0.4
Tape–urethral–lumen distance (Valsalva)	2.6 ± 0.9	2.8 ± 1.1	0.391	−0.6; 0.2
Sagital Tape angle (rest)	132.5 ± 35.7	154.9 ± 25.6	0.002	−31.6; −6.1
Sagital Tape angle (Valsalva)	126.9 ± 40.9	139.2 ± 30.3	0.288	−24.5; 6.2
Tape position (reposo)	55.1 ± 13.4	58.7 ± 12.8	0.236	−9.5; 2.4
Tape position (Valsalva)	51.3 ± 14.5	48.6 ± 15.3	0.410	−3.8; 9.3
Movement concordant between the tape and the urethra				
Concordant	53/63(84.1%)	21/29 (72.4%)	0.302	
Discordant	10/63 (15.9%)	8/29 (27.6%)		−30.3; 6.9
Axial Tape angle (Rest)	139.3 ± 19.0	129.8 ± 18.9	0.029	1.0; 17.9
Axial Tape angle (Valsalva)	145.1 ± 20.2	140.8 ± 22.0	0.485	−5.8; 15.4
LAM avulsión	13/63 (20.6%)	3/29 (10.3%)	0.361	−5.1; 25.7
Levator hiatus area (rest)	17.0 ± 2.9	17.5 ± 3.0	0.520	−1.8; 0.9
Levator hiatus area (Valsalva)	20.8 ± 6.4	21.3 ± 5.0	0.480	−3.0; 1.2

**Table 3:** Ultrasound evaluation of the study population differentiating asymptomatic patients from patients with SUI, UUI, MIU after placement of the SIMS.

	Post–Surgical Clinical Diagnosis				p1	p2	p3
	Asymptomatic (n: 63)	SUI (n: 8)	UUI (n: 12)	MUI (n: 9)			
Bladder neck–symphyseal distance (rest)	23.8 ± 4.9	26.5 ± 3.5	21.1 ± 6.6	24.1 ± 9.1	0.481	0.332	0.998
Bladder neck–symphyseal distance (Valsalva)	15.8 ± 7.4	14.3 ± 12.1	14.3 ± 4.9	15.1 ± 7.5	0.932	0.882	0.989
Posterior urethro–vesical angle (rest)	118.5 ± 17.7	117.2 ± 6.2	114.7 ± 19.4	111.1 ± 10.6	0.995	0.845	0.515
Posterior urethro–vesical angle (Valsalva)	132.2 ± 25.0	146.2 ± 16.5	122.9 ± 17.4	126.7 ± 19.7	0.293	0.493	0.875
Gap between the symphysis pubis–tape (rest)	14.1 ± 4.3	13.4 ± 1.3	15.5 ± 4.0	13.1 ± 2.2	0.945	0.636	0.831
Gap between the symphysis pubis–tape (Valsalva)	12.8 ± 3.2	11.4 ± 1.4	13.7 ± 2.7	12.9 ± 2.2	0.523	0.712	1
Tape–urethral–lumen distance (rest)	3.1 ± 0.8	2.9 ± 0.6	3.8 ± 1.2	2.6 ± 1.0	0.931	0.061	0.346
Tape–urethral–lumen distance (Valsalva)	2.6 ± 0.9	2.8 ± 0.4	3.3 ± 1.5	2.3 ± 0.9	0.908	0.062	0.814
Sagital Tape angle (rest)	132.5 ± 35.7	168.9 ± 10.9	143.3 ± 29.8	157.8 ± 23.6	0.375	0.001	0.025
Sagital Tape angle (Valsalva)	126.9 ± 40.9	136.7 ± 9.1	138.8 ± 37.6	141.9 ± 34.6	0.869	0.694	0.616
Tape position (reposo)	55.1 ± 13.4	63.5 ± 10.8	59.1 ± 13.4	53.8 ± 13.3	0.258	0.704	0.989
Tape position (Valsalva)	51.3 ± 14.5	61.3 ± 9.9	48.1 ± 10.3	37.9 ± 17.3	0.173	0.847	0.026
Movement concordant between the tape and the urethra							
Concordant	53/63 (84.1%)	2/8 (25.0%)	12/12 (100%)	7/9 (77.8%)	0.001	0.349	0.639
Discordant	10/63 (15.9%)	6/8 (75.0%)	0/12 (0%)	2/9 (22.2%)			
Axial Tape angle (Rest)	139.3 ± 19.0	118.3 ± 15.4	133.4 ± 22.7	135.5 ± 12.4	0.003	0.283	0.678
Axial Tape angle (Valsalva)	145.1 ± 20.2	159.1 ± 9.0	133.8 ± 27.3	133.7 ± 11.6	0.034	0.134	0.102
LAM avulsión	13/63 (20.6%)	0/8 (0%)	2/12 (16.7%)	1/9 (11.1%)	0.336	1	0.679
Levator hiatus area (rest)	17.0 ± 2.9	17.3 ± 0.6	18.8 ± 3.0	15.9 ± 3.8	0.996	0.159	0.631
Levator hiatus area (Valsalva)	20.8 ± 6.4	23.1 ± 6.7	21.3 ± 2.3	19.7 ± 5.7	0.675	0.989	0.936

p1: Difference between asymptomatic vs. SUI. p2: Difference between asymptomatic vs. UUI. p3: Difference between asymptomatic vs. MIU.

## Data Availability

The data is maintained by the main author.
